# Facility-Based Maternal Death in Western Africa: A Systematic Review

**DOI:** 10.3389/fpubh.2018.00048

**Published:** 2018-02-26

**Authors:** Nathali Gunawardena, Ghose Bishwajit, Sanni Yaya

**Affiliations:** ^1^Faculty of Science, University of Ottawa, Ottawa, ON, Canada; ^2^School of International Development and Global Studies, University of Ottawa, Ottawa, ON, Canada

**Keywords:** obstetric care, maternity care, access, facility level, barriers, maternal deaths, Western Africa, systematic review

## Abstract

**Background:**

For exploring maternal death, supply and demand-side factors can be characterized by the three delays model developed by Thaddeus and Maine (1994). The model comprises delay in deciding to seek care (delay 1), delay in reaching the health facility (delay 2), and delay in receiving quality care once at the health facility (delay 3). Few studies have comprehensively dealt with the health systems delays that prevent the receipt of timely and appropriate obstetric care once a woman reaches a health facility (phase III delays). The objective of the present study was to identify facility-level barriers in West African health facilities.

**Methods:**

Electronic databases (Medline, cumulative index to nursing and allied health literature, Centre for Agriculture and Biosciences International Global Health, EMBASE) were searched to identify original research articles from 1996 to 2016. Search terms (and synonyms) related to (1) maternal health care (e.g., obstetric care, perinatal care, maternal health services); (2) facility level (e.g., maternity unit, health facility, phase III, hospital); and (3) Western Africa (e.g., Nigeria, Burkina Faso) were combined. This review followed the preferred reporting items for systematic reviews and meta-analyses.

**Results:**

Of the 2103 citations identified, 13 studies were eligible. Studies were conducted in Nigeria, Burkina Faso, Gambia, Guinea, Senegal, and Sierra Leone. 30 facility-level barriers were identified and grouped into 6 themes (human resources, supply and equipment, referral-related, infrastructure, cost-related, patient-related). The most obvious barriers included staff shortages, lack of maternal health services and procedures offered to patients, and lack of necessary medical equipment and supplies in the health-care facilities.

**Conclusion:**

This review emphasizes that phase I and phase II barriers are not the only factors preventing women from accessing proper emergency obstetric care. Health-care facilities in Western Africa are inadequately equipped to handle the obstetric needs of patients. Supply-side barriers must be addressed to reduce maternal mortality in the region.

## Background

Understanding the causes and contextual factors surrounding maternal deaths occurring every year in Africa is important for health programming and policy. Western Africa has among the highest maternal mortality rates in the world with a maternal mortality ratio (MMR; maternal deaths per 100,000 live births) of 679 in 2015 (1). This is much higher than the MMR of Central and Eastern Europe and the Common wealth of Independent States, which had an MMR of 25 in 2015 (1).

Although the number of maternal deaths has been drastically reduced in the last 25 years from an MMR of 1,070 in 1990, Western Africa still fares much worse than other regions in the world in the number of maternal deaths (1). The drop in maternal deaths in Western Africa along with the rest of the world is due in part to countries adopting the Millennial Development Goal 5 (2). The goal aimed to reduce by three quarters, between 1990 and 2015, the MMR and to achieve, by 2015, universal access to reproductive health (3).

Despite adopting Millennial Development Goal 5, the majority of Sub-Saharan countries fall short of the goal (4). Many women in the region are still dying unnecessarily. The World Health Organization (WHO) estimates that at least 88–98% of maternal deaths can be averted with timely access to existing, emergency obstetric interventions (5).

Most maternal deaths occur between the third trimester and first week post-delivery (6, 7). Determining the specific medical causes of maternal death is difficult, especially in cases where the mother gives birth at home (8). Severe bleeding, hypertension, and infections are the primary direct causes of maternal death in Western Africa (9). HIV is also related to pregnancy-related deaths in many hospitals (10, 11), but the exact contribution of HIV/AIDS to maternal deaths is still unknown.

Thaddeus and Maine’s 1994 study examined factors that contribute to maternal mortality between the onset of the obstetric problem and its outcome (12). The study identified three levels of delay that increase maternal mortality rates including delay in deciding to seek care (delay 1), delay in reaching the health facility (delay 2), and delay in receiving quality care once at the health facility (delay 3). The three delays model is extensively used in studies examining maternal mortality. Many studies have examined the demand-side barriers (phase I and phase II delays) since the publication of Thaddeus and Maine’s study (13–15).

Despite these contributions, few studies have examined the supply-side barriers (phase III) that delay women from receiving quality care upon reaching a health-care facility. These barriers, including staff shortages, inadequate supply of drugs and supplies, although neglected in studies, are often important contributors in determining maternal health outcomes.

The following study aims to identify facility-level barriers to the provision of evidence-based maternal health care in West African health-care facilities. Identification and understanding of these barriers can help improve maternal health care in the region in the hopes of reducing maternal deaths.

## Methods

### Search Strategy

A systematic review of English literature was performed on 29th January 2017 to identify primary research articles focusing on facility-level barriers to accessing evidence-based maternal health care in Western Africa published from 1996 to 2016. This time frame was chosen because the “three delays” model was first published in 1994, and because the rapid development of maternal health care in the 1990s reduced the relevance of data from earlier decades. An electronic search was conducted using online databases MEDLINE, EMBASE, cumulative index to nursing and allied health literature (CINAHL), and Centre for Agriculture and Biosciences International (CABI) Global Health. The search strategy is shown in Appendix A and the preferred reporting items for systematic reviews and meta-analyses flow diagram is shown in Appendix B.

### Eligibility Criteria

Articles were included if the title and abstract indicated that they reported the results of original research studies in the English language using quantitative, qualitative, or mixed method approaches. Articles were also required to have been undertaken in Western Africa and to report the association between delays at the facility level and maternal mortality or severe morbidity. Articles were excluded if the research solely examined patient-side or community-level barriers leading to treatment delays, or if only the clinical causes of maternal death were reported. The titles and abstracts of the retrieved articles were screened independently by two authors (Nathali Gunawardena and Sanni Yaya). Eligibility criteria were then applied to the retrieved set of articles by the same authors. A third investigator (Ghose Bishwajit) resolved any differences.

### Data Extraction

An electronic data collection form was developed by one investigator (Sanni Yaya). Upon conducting the three levels of screening, the investigators (Nathali Gunawardena and Sanni Yaya) used the data extraction tool to independently extract the following information from each article: (i) country where the study was conducted in; (ii) type of study (quantitative, qualitative, and mixed); (iii) data collection procedures (self-administered structured questionnaire, focus group, and semi-structured telephone and face-to-face interviews); (iv) sampling strategy; (v) number of facilities/districts covered; (vi) barriers to the provision of evidence-based maternal health care in the facilities; and (vii) attempts made to quantify barriers. Extracted data can be found in Tables A1 and A2 in Appendix C.

## Results

30 facility-level barriers to the provision of evidence-based maternal health care were identified and grouped into 6 themes (human resources, supply and equipment, referral-related, infrastructure, cost-related, patient-related). Barriers were also identified as being explicitly or implicitly stated to map trends in literature. Of the 13 studies, 7 were conducted in Nigeria, 2 in Burkina Faso, and 1 each in Gambia, Guinea, Senegal, and Sierra Leone. Seven studies used quantitative research methods, three used qualitative research methods, and three used a mixed methods approach.

### Human Resources

Human resources-related barriers were reported in 10 out of 13 papers. The most widely reported human resource-related barrier that was cited in 9 out of the 10 papers was the shortage of staff at health-care facilities. In one study conducted at a Nigerian hospital, there was an insufficient number of nursing and midwifery staff to cover the large number of obstetric cases in the hospital (16). Another common human resource-related barrier was inadequately trained staff, with 6 out of the 10 studies reporting the issue. In one study conducted at the University of Calabar Teaching Hospital in Nigeria, 66% of 132 non-physician obstetric health-care providers at the hospital had used a partograph, a simple tool that is designed to detect early signs of abnormal progress and can reduce incidence of complication in labor, yet only 13.6% of these health-care workers were confident in using the tool (17). In another study conducted in Sierra Leone, anesthetists and pediatricians were scarce in the health-care facilities, and many facility births in the country were found to be conducted by traditional birth attendants, maternal and child health aides, and state-enrolled community health nurses who may not meet the definition of a skilled birth attendant (18). 4 out of the 10 articles citing human resources-related barriers noted unfriendly staff or poor quality of care provided by staff as a barrier. In a study conducted in Nigeria, 70.8% of women reported unfriendly attitude of staff at the health-care facility as a reason for the non-utilization of health facility for delivery (19).

### Supply/Equipment-Related Barriers

Supply and equipment-related barriers were reported in 10 out of the 13 studies (16, 18, 20–27). 8 out of the 10 studies reported lack or failure of equipment or supplies at the health-care facilities as a barrier to receiving evidence-based maternal health care (16, 18, 20, 23–27). In one study conducted in Senegal, a series of risk factors related to the functioning of the health system were identified and among these risk factors, failure of medical equipment had a high odds ratio (OR = 55.0) (23). The other supply/equipment-related barrier found in the studies included the lack of blood and medication at the health-care facilities. This barrier was cited in 6 of the 10 studies (16, 18, 21, 22, 26, 27). In one study in Gambia, obtaining blood for transfusion was found to be a difficult process (21). One man in the study describes the taxing and time consuming process of trying to find blood for his wife who needed a transfusion. He describes the ordeal saying “At the ward I was told to find two bottles of blood for her (referring to his wife). I went to the lab to look for blood but was told (by laboratory staff) that there was none (…). I decided to donate but my blood (group) was different. It was already night so I went home (…). In the evening of the following day a friend came to donate her one bottle.” In this same study, patients describe a shortage of drugs at their hospital and having to go to find medications at pharmacies, medications they sometimes could not afford.

### Referral-Related Barriers

4 out of 13 studies cited referral-related barriers (16, 19, 24, 26). In one study conducted in Nigeria, lack of transport, fear of the unknown, lack of emotional support, and unfamiliar environment were the common reasons reported by patients when citing why they avoided referrals (26). In another study conducted in Southern Nigeria, the most common type of type III delay that was reported was delayed referral from private hospitals (16).

### Infrastructure-Related Barriers

2 out of 13 studies cited infrastructure-related barriers (18, 28). In one study conducted in Nigeria, most of the visited health-care facilities did not have a guaranteed power supply whenever there were obstetric emergencies (28). In another study in Sierra Leone, only 10% of hospitals and community health centers had regular electricity, only 60% had a water supply, and there was running water and functioning toilets in only 44 and 67% of community health centers and hospitals, respectively (18).

### Cost-Related Barriers

2 out of 13 studies cited cost-related barriers (18, 19). In a study conducted in Sierra Leone, pregnant women were made to pay fees that varied widely depending on the health-care facility. The fees were arbitrary and families could not predict the out of pocket expenditure. These fees were charged despite national policy stating that maternal and child health services are free (18). In a study conducted in Nigeria, lack of money for transportation was cited as a barrier (19).

### Patient-Related Barriers

2 out of 13 studies cited patient-related barriers (25, 26). In a study conducted in the health district of Bogandé, Gnagna province in the Eastern region of Burkina Faso, through interviews with health-care workers, it was identified that a difficulty faced in the provision of good quality antenatal care was the refusal of some women to be examined by male health-care workers (25). In one study in Nigeria, some female patients had to be referred to other health-care facilities due to their sociocultural preferences (26).

## Discussion

The current study identified facility-level barriers to the provision of evidence-based maternal health care in Western Africa. 13 articles that examine phase III delays in maternal health care in Western Africa were found by systematically searching 4 electronic databases. The most commonly cited barriers included shortage of staff, poorly trained staff, lack or failure of equipment, poor quality of care or unfriendly attitude provided by staff, and insufficient drug and blood supply.

The most commonly cited barrier noted in the studies was human resources-related barriers, especially staff shortages. The largest gaps between requirements of the health-care facilities and staff in Sub-Saharan Africa are for nurses and midwives (29). Changes in the number of staff in the Sub-Saharan region exist for various different reasons including inflows from training, immigration and employment; employment status; and outflow due to death, retirement, and emigration (29). In a study analyzing maternal mortality, the density of human resources (doctors, nurses) in a region was found to be significant in determining the maternal mortality rate (30). The study illustrated that having a sufficient number of medical staff in a region is essential in reducing maternal mortality. Staff shortages do not only have a direct impact on patient care, but shortages can have indirect effects as well by lowering the job satisfaction of the existing workforce. Teamwork between medical staff is an essential component of patients receiving high-quality maternal health care. Teamwork allows interactions between colleagues, and mutual support which contribute to job satisfaction (31). The absence of team members can reduce job satisfaction and morale of maternal health workers resulting in lower quality of care for patients (32). Staff shortages need to be addressed in order to improve maternal health outcomes. WHO estimates that Sub-Saharan Africa needs a substantial increase in the number of health-care professionals with midwifery skills to meet universal coverage for maternal health (33).

One of the 13 studies reviewed compared the impact of phase III delays on maternal health outcomes to the impact of phase I and phase II delays. Phase III delays were found to be the most frequent cause of maternal deaths, occurring in 61.9% of cases in the hospital. Phase I delay occurred in 28.6% of cases, and there were no recorded cases of phase II delays (16).

### Strengths and Limitations

This systematic review has several different limitations. The first limitation is that data sources for the study were published in four online databases including Medline, CABI Global Health, CINAHL, and EMBASE. Only studies published in the English language were used for the review. Due to these reasons, there is a chance that other relevant studies describing facility-level barriers to receiving maternal health care may have been missed for the reason that they may have been published elsewhere or in a language other than English.

Another limitation of the review is that several studies failed to examine barriers from the perspective of health-care workers and primarily focused on barriers from the point of view of the patients. In only three studies, health-care providers were interviewed (16, 17, 25). Due to this reason, some barriers may have been missed.

Study bias is another potential limitation of the review. Authors of the studies included in the review may have focused on certain barriers over others based on their area of interest. As a result, the frequency of barriers presented in the review may be based on the author’s area of interest instead of the actual frequency of barriers occurring in Western Africa.

Studies included in this review were conducted in specific West African countries (Nigeria, Burkina Faso, Gambia, Guinea, Senegal, and Sierra Leone). No studies could be found that studied facility-level barriers in other West African nations. Although it is likely that the same barriers found in this review exist in other West African nations, additional phase III barriers may exist in these countries. In addition to this, the majority of the included studies used in this review that resulted from the data base search were conducted in Nigeria (7 out of 13). This may introduce bias because some of the barriers that we have generalized for Western Africa may in reality be specific to Nigeria.

Although we have stated several causes of facility-based maternal mortality in Western Africa, these causes may not be conclusive as postmortems are rarely carried out in Africa on maternal deaths (34).

Finally, barriers identified from the articles are difficult to compare as a wide range of sampling strategies, data collection methods, and study designs were used in the different papers.

### Implications and Recommendations

The studies included in this review display the large role that facility-level barriers play in maternal health-related complications. This review highlights the importance of addressing these phase III barriers to improve maternal health without solely focusing on the demand-side barriers.

One of the most common barriers addressed in the reviewed articles included staff shortages. Allowing part-time work, more flexible work schedules, contracting retired staff, and offering financial incentives for working on holidays or for overtime work are just some of the ways that local hospitals and health-care facilities can maintain steady staff numbers. These methods proved successful in Gambia and Ghana (29). Not only do the number of maternal health professionals in health-care facilities need to increase, but the quality of care provided by these health-care workers needs to as well. Quality of care received by mothers and babies in developing countries is often reported as poor, yet this important factor is often overlooked compared to barriers to access to care (35). In order to improve maternal health outcomes, and to ensure that women are more likely to access needed health-care services, investment in the quality of health posts is a priority (36).

Efforts should also be made to come up with replicable methods to assess facility-level barriers in various settings so that comparisons between barriers can be made more easily. At present, there are no methodologies to make comparisons between barriers in different countries that are widely accepted (37).

## Conclusion

Many facility-level barriers to the provision of evidence-based maternal health care exist in Western Africa that result in countless deaths that could otherwise be prevented. Many studies do not address these facility-level barriers and instead focus on demand-side barriers making it difficult to make improvements in maternal health outcomes in the region. It is important for future studies to address both types of barriers to prevent unnecessary deaths. Addressing the barriers discussed in this review along with finding better methods of identifying more barriers and to make comparisons between them should be seen as a focus for future studies.

## Availability of Data and Materials

All data generated or analyzed during this study are included in this published article.

## Author Contributions

NG and SY conceived of the study, participated in its design and coordination, and drafted the manuscript. GB resolved any differences in applying the eligibility criteria to the articles and critically reviewed the manuscript for its intellectual content. All authors read and approved the final manuscript.

## Conflict of Interest Statement

The authors declare that the research was conducted in the absence of any commercial or financial relationships that could be construed as a potential conflict of interest.

## Figures and Tables

**Figure A1:**
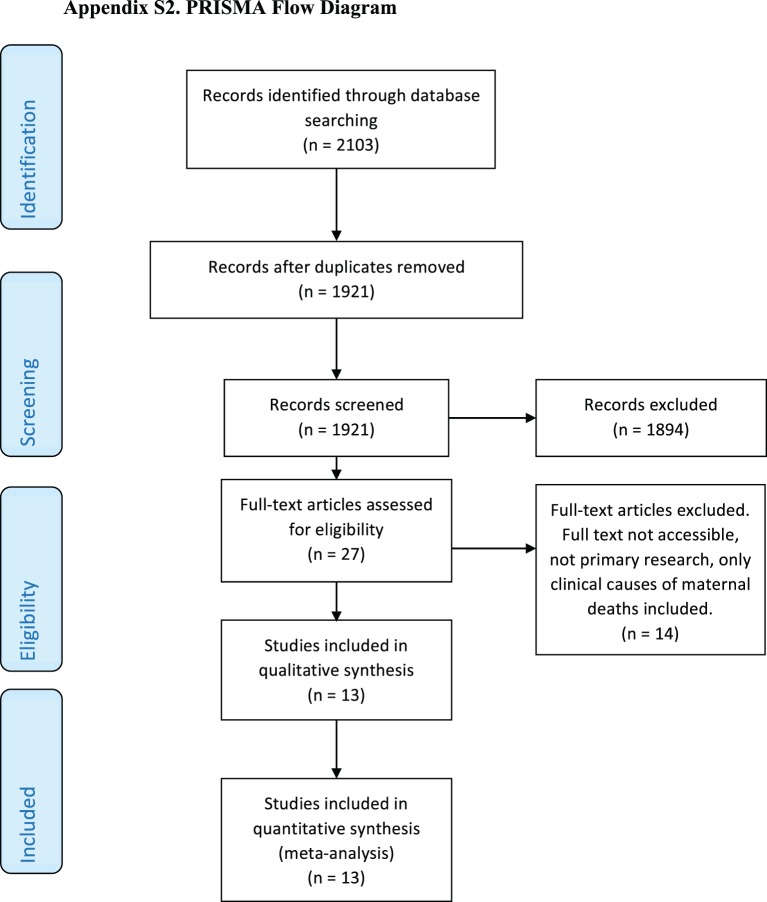
PRISMA flow diagram (38).

## Abbreviations

CINAHL, cumulative index to nursing and allied health literature; CABI, Centre for Agriculture and Biosciences International; MMR, maternal mortality ratio; PRISMA, preferred reporting items for systematic reviews and meta-analyses; WHO, World Health Organization.

## Appendix A

### Search Strategy Example (Ovid Medline)

1. exp Maternal Health Services
2. exp Perinatal Care/
3. Obstetrics/
4. (Maternal Health Services).tw.
5. Obstetrics.tw.
6. (Perinatal Care).tw.
7. 1 or 2 or 3 or 4 or 5 or 6
8. Health Facilities/
9. Hospitals/
10. (Health Facilities).tw.
11. Hospital.tw.
12. Hospitals.tw.
13. (Maternity Unit).tw.
14. (Phase III).tw.
15. (Facility Level).tw.
16. 8 or 9 or 10 or 11 or 12 or 13 or 14 or 15
17. Western Africa.tw.
18. exp Africa, Western/
19. Benin.tw.
20. Burkina Faso.tw.
21. Cape Verde.tw.
22. Gambia.tw.
23. Ghana.tw.
24. Guinea.tw.
25. Guinea-Bissau.tw.
26. Ivory Coast.tw.
27. Liberia.tw.
28. Mali.tw.
29. Mauritania.tw.
30. Niger.tw.
31. Nigeria.tw.
32. Senegal.tw.
33. Sierra Leone.tw.
34. Togo.tw.
35. 17 or 18 or 19 or 20 or 21 or 22 or 23 or 24 or 25 or 26 or 27 or 28 or 29 or 30 or 31 or 32 or 33 or 34
36. 7 and 16 and 35
37. Limit 36 to (English language and yr = “1996–2016”)

## Appendix C

### Extracted Data

**Table A1:** Characteristics of included studies.

Reference	Location	Design	Number of facilities/districts covered	Data collection	Sampling strategy	Attempts to quantify barriers
Agan et al. (17)	Nigeria	Quantitative, cross sectional study	1 facility/1 district	Semi-structured, self-administered questionnaire	Purposive sampling	Yes
Baguiya et al. (20)	Guinea	Quantitative, cross sectional study	502 facilities	Data taken from registries (national cross-sectional census of health facilities), interviews (head of units and key health workers)	Survey sampling	Yes
Cham et al. (21)	Gambia	Qualitative	1 facility/1 district	Interviews	Purposive sampling	No
Georges Dayitaba et al. (22)	Burkina Faso	Quantitative	52 facilities/53 districts	Interviews	Probability sampling	Yes
Daniel et al. (28)	Nigeria	Qualitative	121 facilities	Interviews, cross sectional survey	Random sampling	No
Garenne et al. (23)	Senegal	Qualitative	3 facilities/1 district	Case–control study	Random sampling	Yes
Mezie-Okoye et al. (24)	Nigeria	Quantitative, cross sectional study	12 facilities/1 district	Cross sectional facility-based survey, key informant interviews (semi-structured questionnaire/checklist)	Stratified sampling	Yes
Moore et al. (19)	Nigeria	Quantitative, cross sectional study	6 facilities/1 district	Cross-sectional questionnaire	Multistage sampling (selection of the health centers), simple random sampling (to select the subjects)	Yes
Nikiema et al. (25)	Burkina Faso	Quantitative and qualitative	17 facilities/1 district	Facility-level cross sectional audit of equipment, number of staff, etc. Non participating observation (for all the operations, attitudes, and questions put to the pregnant woman during ANC) Scheduled interview with team leader using semi-structured questionnaire	Probability sampling	No
Nnebue et al. (26)	Nigeria	Quantitative and qualitative, cross sectional study	4 facilities/1 district	Client exit interviews, observation checklist, key informant interviews	Multistage sampling technique	Yes
Okoli et al. (27)	Nigeria	Quantitative	652 facilities	Survey with cross sectional descriptive design, semi-structured interview method (of the managers of the health-care facilities)	Survey sampling	Yes
Omo-Aghoja et al. (16)	Nigeria	Qualitative and quantitative	1 facility/1 district	Review of maternity death records, computer records, clinical audits and monthly reviews Objective review of the status of the facilities in terms of care (availability, adequacy and quality) interviews	Probability sampling	Yes
Oyerinde et al. (18)	Sierra Leone	Quantitative, cross sectional study	145 facilities	Cross sectional survey	Systematic random sampling	Yes

**Table A2:** Barriers to evidence based maternal health care.

Reference, country	Human resources	Supply/equipment	Referral-related	Infrastructure	Cost-related	Patient-related
Agan et al. (17), Nigeria	Non availability and shortage of man power, little knowledge by health care givers on how to use the health care equipment (partograph), little time for the health care givers to use necessary equipment (high workload) All barriers explicit					
Baguiya et al. (20), Guinea	Lack of training for staff (explicit), staff shortage (implicit)	Lack of equipment (explicit)				
Cham et al. (21), Gambia		Lack of blood, shortage of medication (explicit)				
Georges Dayitaba et al. (22), Burkina Faso	Lack of physicians with surgical skills, surgical assistants, and anesthesiologist assistants (explicit)	Lack of blood (explicit)				
Daniel et al. (28), Nigeria	Lack of doctors (explicit)			Lack of proper power supply (explicit)		
Garenne et al. (23), Senegal	Lack of available personnel at time of admission (explicit)	Medical equipment failure (explicit)				
Mezie-Okoye et al. (24), Nigeria	Lack of sufficient number of trained staff (explicit)	Lack of sufficient number of equipment and supplies (explicit)	Lack of transportation for referral (explicit)			
Moore et al. (19), Nigeria	Unfriendly attitude of staff (explicit), unavailability of staff (explicit), lack of urgency at facility (explicit)		Long distance to health-care facility (explicit), unavailability of means of transportation (explicit)		Lack of money for transportation (explicit)	
Nikiema et al. (25), Burkina Faso	Poor quality of care provided (explicit), staff shortage (implicit), procedures not followed by staff (implicit)	Lack of equipment (implicit)				Refusal by women to be examined by male health workers (implicit)
Nnebue et al. (26), Nigeria	Lack of skilled personnel (explicit), poor quality of care by staff (explicit)	Lack of drugs and supplies (explicit), lack of equipment (explicit)	Poor (lack of) referral (explicit)			Sociocultural preferences (explicit)
Okoli et al. (27), Nigeria		Lack of a sufficient amount of supplies or drugs (explicit)				
Omo-Aghoja et al. (16), Nigeria		Lack of blood and oxygen available at hospital (explicit), lack of necessary back up equipment (explicit)	Delayed referral from private hospitals (explicit)			
Oyerinde et al. (18), Sierra Leone	Poorly trained staff (implicit), insufficient number of trained staff available (explicit), poor quality of care (explicit)	Insufficient equipment, drugs and supplies (explicit)		Lack of plumbing, electricity, running water	Cost (explicit)	
Total	10	10	4	2	2	2
